# POSH undergoes phase separation and co-condensation with SHANK2/3 to regulate spine development

**DOI:** 10.1093/procel/pwaf066

**Published:** 2025-08-04

**Authors:** Minghui Yao, Ling Yuan, Yu Zheng, Zhiheng Xu

**Affiliations:** State Key Laboratory of Molecular Developmental Biology, Institute of Genetics and Developmental Biology, Chinese Academy of Sciences, Beijing 100101, China; University of Chinese Academy of Sciences, Beijing 100101, China; Center for Medical Genetics & Hunan Key Laboratory of Medical Genetics, School of Life Sciences, Central South University & MOE Key Lab of Rare Pediatric Diseases, Changsha 410083, China; State Key Laboratory of Molecular Developmental Biology, Institute of Genetics and Developmental Biology, Chinese Academy of Sciences, Beijing 100101, China; University of Chinese Academy of Sciences, Beijing 100101, China; State Key Laboratory of Molecular Developmental Biology, Institute of Genetics and Developmental Biology, Chinese Academy of Sciences, Beijing 100101, China; University of Chinese Academy of Sciences, Beijing 100101, China

## Dear Editor,

The Shank gene family (*SHANK1*, *SHANK2*, and *SHANK3*) comprises high-risk genetic contributors to autism spectrum disorders (ASD) (Durand et al., 2007; Monteiro and Feng, 2017). Copy-number variants and truncating mutations in these genes have been identified in ~1% of ASD patients (Leblond et al., 2014; Moessner et al., 2007). Research in *Shank*-deficient mouse models demonstrates that restoring *Shank* expression in adulthood can enhance synaptic protein levels, correct defects in synaptic morphology and function, and improve ASD-related behavioral deficits (Guo et al., 2019; Mei et al., 2016). These findings suggest that targeting the postsynaptic function of SHANK proteins may be a promising therapeutic strategy for ASD.

Synaptic localization of SHANK2 and SHANK3 depends on their conserved C-terminal domains, including the proline-rich domain and sterile alpha-motif (SAM) domain (Boeckers et al., 2005). The SAM domain enables SHANK proteins to bind Zn²⁺ and be recruited to the postsynaptic density (PSD) in a zinc-dependent manner (Baron et al., 2006). Notably, Zn²⁺ levels have a greater impact on the synaptic localization of SHANK3 than SHANK2. Moreover, zinc deficiency specifically disrupts SHANK2 postsynaptic localization in the cortex but not in the striatum, hippocampus, or cerebellum, indicating additional mechanisms beyond zinc-induced assembly that regulate SHANK targeting (Grabrucker et al., 2014).

*POSH* (plenty of SH3s, also named *SH3RF1*) is another high-risk ASD gene (Satterstrom et al., 2020). Our previous work has shown that *Posh* deficiency reduces SHANK2/3 abundance in PSD and impairs dendritic spine development (Yao et al., 2022). *Posh* cKO mice exhibit autistic-like behaviors and learning and memory deficits, resembling *Shank2*/*3* deletion/mutation models. However, the mechanisms organizing autism-associated proteins at the PSD and regulating synaptic development remain unclear.

**Figure 1.**
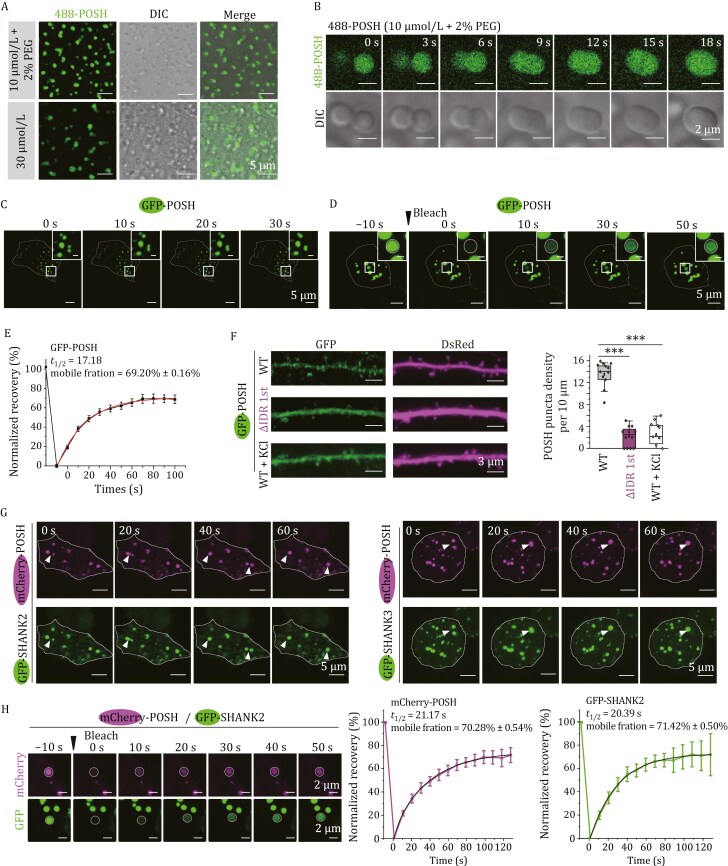
POSH undergoes phase separation and co-condenses with SHANK2/3. (A) Representative images of POSH droplets (10 µmol/L) formed in a buffer solution containing 2% PEG 8000 or 30 µmol/L of POSH alone. Scale bars: 5 µm. (B) Time-lapse images showing POSH droplet fusion events *in vitro*. Scale bars: 2 µm. (C) Time-lapse images showing the fusion of GFP-POSH puncta in HEK293T cells. Whole cell images scale bars: 5 μm; magnified boxed regions scale bars: 1 μm. (D and E) FRAP analysis of GFP-POSH condensates in HEK293T cells. (D) Representative images of GFP-POSH condensates before photobleaching (Pre-bleach) and immediately after photobleaching (Post-bleach). White circles denote photobleached regions. Scale bars: Whole-cell image, 5 µm; magnified inset, 1 µm. (E) Quantification of fluorescence recovery from the FRAP analysis. Data are presented as the mean ± SEM (*n* = 25 puncta). The red curve corresponds to a double exponential fit of the data. Key parameters include a recovery half-time (*t*_1/2_) of 17.18 s and a mobile fraction of 69.20%, calculated from the recovery curve plateau. (F) Hippocampal neurons from *Posh* cKO mice were transfected with DsRed and either GFP-POSH or ΔIDR 1st, with or without KCl stimulation. POSH condensates were identified using Imaris 3D rendering. Quantitative analysis of POSH puncta density (sphericity > 0.8) was performed. *n* = 12–15 neurons from three independent experiments per group. Data are presented as boxplots (centerline: median; box limits: Q1, Q3; whiskers: min/max within 1.5× IQR of Q1/Q3). ***P < 0.001, one-way ANOVA with Tukey’s test. (G) Time-lapse images showing the fusion of mCherry-POSH condensates with GFP-SHANK2 or GFP-SHANK3 puncta in HEK 293T cells (indicated by arrows). Scale bars: 5 μm. (H) FRAP analysis of mCherry-POSH and GFP-SHANK2 within co-condensates. Representative images show co-condensates before bleaching (Pre-bleach) and immediately after photobleaching (Post-bleach). White circles denote photobleached regions. Normalized fluorescence recovery curves for mCherry-POSH (magenta) and GFP-SHANK2 (green) are plotted. For each co-condensate, the mCherry and GFP channels were independently bleached and analyzed to evaluate the dynamics specific to each component. Data are presented as the mean ± SEM (*n* = 7 co-condensates). The black curve corresponds to a double exponential fit of the data. Scale bars: 2 μm. Experiments in (A–D, G and H) were repeated three times.

Sequence analysis predicted multiple intrinsically disordered regions (IDRs) in POSH (Fig. S1A). We therefore investigated whether POSH has an intrinsic ability to phase separate. Purified POSH (10 µmol/L) from *E*. *coli* formed droplets in physiological salt buffer with 2% PEG or at 30 µmol/L without crowding agent (Fig. 1A). We then constructed a phase diagram by mixing POSH and PEG concentrations and detecting liquid droplets via phase-contrast microscopy (Fig. S1B). Live cell imaging showed that POSH condensates dynamically fused (Fig. 1B). In HEK293T cells, EGFP-POSH formed condensates/puncta (approximately 10% of cells) or a gel-like structure (Movie S1) with condensates exhibiting fusion (Fig. 1C) and rapid fluorescence recovery after photobleaching (FRAP) (Fig. 1D and 1E), which are hallmarks of liquid–liquid phase separation (LLPS). The majority of its fluorescence signal (69.20% ± 0.16%) recovered with a characteristic recovery time of 17.18 s, indicating that POSH is highly dynamic, with rapid exchange of molecules between the droplets and the surrounding solution.

Truncation mapping revealed the IDR (aa 57–139, IDR 1st) as essential for POSH LLPS, while individual SH3 or other IDR deletions had minimal effects (Fig. S1C). In primary cultured hippocampal neurons, GFP-POSH WT formed dendritic puncta, whereas ΔIDR 1st showed diffuse staining (Figs. 1F and S1D). Acute depolarization (90 mmol/L KCl, 5 min) significantly reduced puncta formation (Fig. 1F, WT: 13.41 ± 0.64; ΔIDR 1st: 2.17 ± 0.52; KCl: 2.61 ± 0.58) (Fig. 1F), linking phase separation to synaptic activity.

Given POSH interacts with SHANK2/3 (Yao et al., 2022), we investigated their co-condensation. Co-expressed GFP-SHANK2/3 and mCherry-POSH spontaneously formed highly enriched puncta (Fig. S2A) that fused rapidly (Fig. 1G). In co-condensates containing both mCherry-POSH and GFP-SHANK2, we selectively photobleached mCherry fluorescence to quantify the recovery kinetics of POSH (*t*_1/2_ = 21.17 s, mobile fraction = 70.28% ± 0.54%) and GFP fluorescence to quantify the recovery kinetics of SHANK2 (*t*_1/2_ = 20.39 s, mobile fraction = 71.42% ± 0.50%) (Fig. 1H). These parallel measurements demonstrate that both components exhibit similar fluidity within the shared condensate environment. Notably, in co-condensation with POSH, SHANK2 exhibits significant alterations in its molecular dynamics (Fig. S2B). SHANK2 significantly increased POSH puncta formation (Fig. S2C, POSH alone: 3.47% ± 1.01%; POSH and SHANK2: 21.00% ± 2.56%), while POSH ΔIDR 1st (impaired LLPS) or ΔSH3 1st (impaired SHANK binding) reduced the co-condensation (Fig. S2C, WT: 21.00% ± 2.56%; ΔIDR 1st: 2.67% ± 0.90%; ΔSH3 1st: 8.63% ± 2.60%; Δaa 292–362: 18.54% ± 5.63%; Δaa 363–458: 15.65% ± 2.96%).

Co-immunoprecipitation (Co-IP) identified SHANK2 aa 893–962 as the POSH-binding region (Fig. S2D). Droplet assays revealed SHANK2 recruitment to POSH puncta required both this region and the SAM motif (aa 1,164–1,262) (Fig. S2E, SHANK2 aa 823–1,262: 66.45% ± 7.82%; aa 893–1,262: 80.69% ± 8.91%; aa 944–1,262: 36.11% ± 1.776%; aa 962–1,262, aa 1,164–1,262, aa 823–1,163: hardly detected). The SHANK3 SAM domain forms large sheets composed of helical fibers (Baron et al., 2006), suggesting self-assembly of SHANK3 synergizes with POSH LLPS for co-condensation.

**Figure 2.**
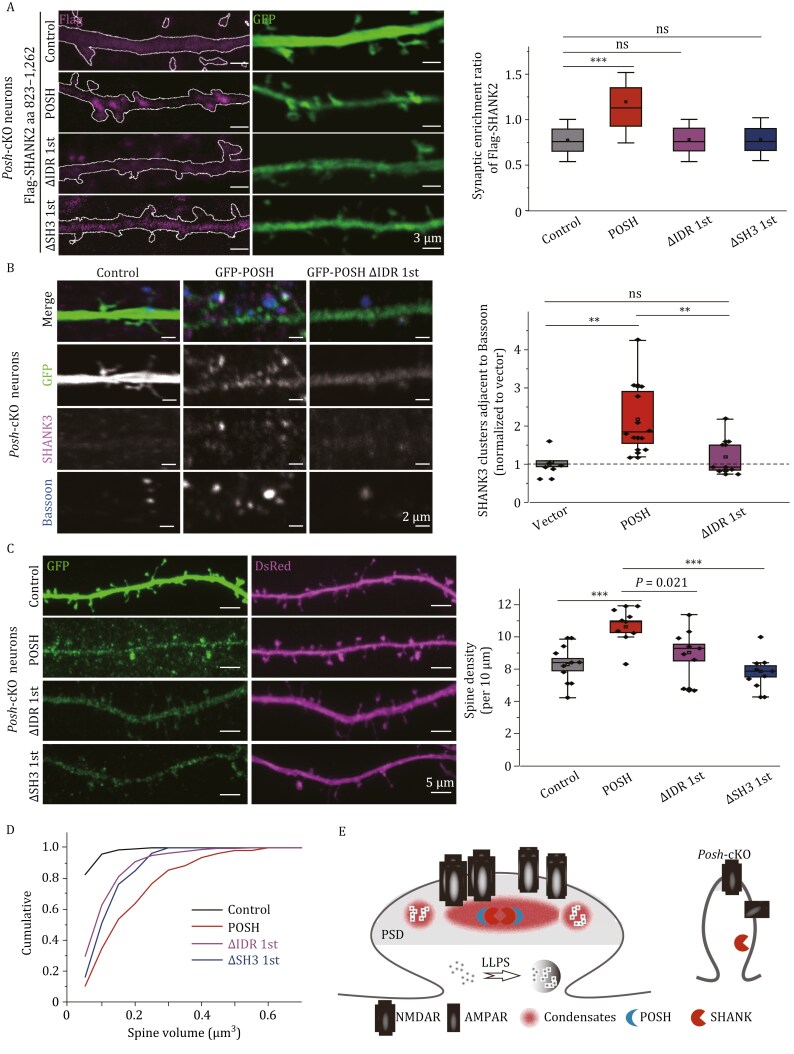
POSH/SHANK co-condensation regulates SHANK synaptic targeting and synaptogenesis. (A) Hippocampal neurons from *Posh* cKO mice were transfected with GFP and Flag-SHANK2 together with empty vector or POSH variants. Imaging data quantification shows the synaptic targeting of Flag-SHANK2 in each group. The synaptic enrichment ratio of Flag-SHANK2 is defined as: ([Flag spine/Flag shaft]/ [GFP spine/GFP shaft]). *n* ≥ 50 dendrites from 12–15 neurons across 3 independent experiments per group. Scale bars, 3 μm. (B) Hippocampal neurons from *Posh* cKO mice were transfected with empty vector, GFP-POSH, or POSH ΔIDR 1st and triple-stained with SHANK3, Bassoon, and GFP antibodies. Quantification shows the density of SHANK3 clusters adjacent to Bassoon, normalized to the vector group. *n* = 12–15 neurons from 3 independent experiments per group. Scale bars, 2 μm. (C) Hippocampal neurons from *Posh* cKO mice were transfected with DsRed together with empty vector, GFP-POSH, ΔIDR 1st, or ΔSH3 1st. Data represent spine density (spines/10 μm dendrite) from *n* = 12–16 neurons per condition across 3 independent experiments. Scale bars, 5 μm. (D) Cumulative distribution of spine volume for the indicated conditions. *P* = 0.0049 (POSH group vs. vector group); *P* = 0.15493 (POSH-ΔIDR 1st group vs. vector group); *P* = 0.0949 (POSH-ΔSH3 1st group vs. vector group); *P* = 0.02952 (POSH-ΔIDR 1st group vs. POSH group); *P* = 0.03786 (POSH-ΔSH3 1st group vs. POSH group). Two-sample Kolmogorov-Smirnov test. (E) A model illustrating the clustering of POSH and SHANK2/3 condensates, which ensures the synaptic localization of SHANK2/3 and subsequent synapse formation regulation. Three factors contribute to the phase formation of POSH and SHANK2/3 condensates: the intrinsically disordered region of POSH, the interaction between POSH and SHANK2/3, and the self-assembly of SHANKs mediated by the SAM domain. PSD, postsynaptic density; cKO, conditional knockout. Panels (A–C) share the same data analysis method. Data are presented as boxplots (centerline: median; box limits: Q1, Q3; whiskers: min/max within 1.5× IQR of Q1/Q3). **P < 0.01; ***P < 0.001; ns, not significant. One-way ANOVA with Tukey’s test.

*Posh* deficiency has been shown to result in reduced level of SHANK2/3 in the PSD fraction (Yao et al., 2022). We characterized and confirmed that the synaptic enrichment of SHANK2 in cultured neurons depended on aa 892–962 and the SAM domain (Fig. S3) (GFP-SHANK2 aa 823–1,262 (1.99 ± 0.26), aa 892–1,262 (1.79 ± 0.12), aa 962–1,262 (0.93 ± 0.05), aa 1,164–1,262 (0.77 ± 0.06) and aa 823–1,163 (0.95 ± 0.08)), aligning with the co-condensation of SHANK2 with POSH observed in HEK293T cells (Fig. S2D). Rescue experiments in *Posh*-cKO neurons showed that POSH WT, but not ΔIDR 1st or ΔSH3 1st, restored synaptic SHANK2 localization (Fig. 2A, control: 0.77 ± 0.02; WT 1.20 ± 0.04; ΔIDR 1st: 0.78 ± 0.03; ΔSH3 1st: 0.78 ± 0.03). Similarly, POSH WT, but not ΔIDR 1st, significantly rescued SHANK3 synaptic clustering as assessed by presynaptic marker (Fig. 2B, vector: 1.00 ± 0.13; POSH: 2.18 ± 0.27; ΔIDR 1st: 1.19 ± 0.16). These results underscore the importance of co-condensation with POSH in the synaptic targeting of SHANK2/3.

We next explored the role of POSH and SHANK2/3 co-condensation in synaptogenesis. In *Posh*-cKO neurons, POSH WT, but not POSH ΔIDR 1st or POSH ΔSH3 1st, significantly increased spine density (Fig. 2C, GFP: 6.23 ± 0.38; WT: 8.41 ± 0.45; ΔIDR 1st: 7.03 ± 0.51; ΔSH3 1st: 5.86 ± 0.35) and restored spine volume (Fig. 2D), indicating co-condensation regulates spine development. We propose a model where POSH phase separation and its co-condensation with SHANK2/3 promote the synaptic targeting of SHANK2/3, thereby regulating PSD organization and spine development (Fig. 2E).

While previous work established that multivalent PSD complexes (e.g., SHANK3-SAPAP3-PSD-95-Homer) can form PSD-like condensates through cooperative interactions, none of their individual components can undergo intrinsic LLPS alone (Zeng et al., 2018). Our study demonstrates that POSH alone can undergo LLPS, driven by the cooperation between its IDR 1st and tandem SH3 domains (Fig. S4A). Notably, POSH’s IDR 1st contains both canonical (XPxXP) and non-canonical (RxxK) proline-rich motifs (PRMs) (Fig. S4B), which mediate multivalent interactions with the SH3 domains to drive condensation (Sieme et al., 2024). Deletion of non-canonical (RxxK) in POSH leads to notably reduced molecular diffusion and lower mobile fraction in POSH condensates, indicating decreased condensate fluidity (Fig. S4C, POSH: *t*_1/2_ = 16.99 s, mobile fraction = 67.18% ± 0.50%, Δaa 74–88 PRM (RxxK): *t*_1/2_ = 24.37 s, mobile fraction = 44.26% ± 0.55%). In contrast, canonical (XPxXP) deletion has no significant impact (Fig. S4C, Δ aa 118–139 PRM (XPxXP): *t*_1/2_ = 16.48 s, mobile fraction = 70.97% ± 0.79%). This shows that the interplay between non-canonical (RxxK) PRMs in POSH’s IDR 1st and SH3 domains regulates POSH’s LLPS.

POSH recruits its binding partner SHANK2/3 into co-condensates, mediated by conserved SHANK2/3 PRMs (PPVPPKP) essential for this incorporation (Fig. S4D). In contrast, PSD-95, a POSH binding partner lacking PRMs, fails to incorporate into POSH condensates (Fig. S4E). This selective recruitment suggests a possible mechanism that weak, multivalent PRM-SH3 interactions may play a crucial role in determining condensate composition. Thus, POSH’s IDR1-driven LLPS provides a unique nucleation mechanism for synaptic condensates, distinct from previous models based on multi-protein complex assembly.

When SHANK2 co-condensed with POSH, it displayed similar molecular dynamics to POSH (Fig. S2B). Both POSH and SHANK exhibit high fluidity in co-condensates (Fig. 1H, mobile fraction: 70.28% ± 0.54% for POSH, 71.42% ± 0.50% for SHANK2), enabling rapid recruitment of downstream effectors and enhanced responsiveness to synaptic signals. This not only provides a mechanism for modulating PSD assembly but also offers a molecular dynamics basis for understanding *SHANK*-mutation-induced synaptic defects in autism.

Emerging evidence suggests that zinc-dependent modulation of SHANK2/3 synaptic localization can ameliorate NMDAR hypofunction and rescue social behavioral deficits in preclinical models of ASD (*Shank2*^−/−^, *Shank3*^−/−^, *Shank3*^ex13–16−/−^ and *Tbr*^+/−^) (Fourie et al., 2018; Lee et al., 2015). Our study shows that co-condensation of POSH and SHANK2/3 critically regulates synaptic SHANK targeting via liquid-phase assembly. This offers a novel compensatory mechanism for *SHANK* haploinsufficiency—by enhancing the efficiency of weak multivalent interactions, it restores synaptic scaffold plasticity and signal integration capacity.

In summary, POSH-mediated LLPS represents a novel mechanism for SHANK2/3 synaptic organization and synapse development. These findings provide new insights into ASD pathogenesis and a potential therapeutic strategy targeting co-condensation.

## Supplementary Material

pwaf066_Supplementary_Figures_S1-S4

pwaf066_Supplementary_Movies_S1

## Footnotes

We express our gratitude to Dr. Mingjie Zhang for his insightful advice. This study was supported by grants from the National Natural Science Foundation of China (NSFC) (32330038, 32394030) and the Ministry of Science and Technology (STI2030-2021ZD0202300).

Z.X. and M.Y. conceived and supervised the project. M.Y. conducted most of the experiments and analyzed the results. L.Y. and Y.Z. provided technical assistance in the phase separation assays. M.Y. and X.Z. wrote the manuscript. All the authors discussed the results and commented on the manuscript.

The authors declare that they have no competing interests.

All experimental procedures involved were performed in accordance with protocols approved by the Institutional Animal Care and Use Committee at the Institute of Genetics and Developmental Biology (IGDB), Chinese Academy of Sciences.

All materials and data reported in this paper will be shared by the corresponding author, Zhiheng Xu, upon request.
