# Supplementary material for: POSH undergoes phase separation and co-condensation with SHANK2/3 to regulate spine development
Source: Protein Cell. 2025 Aug 4;17(1):77–82. doi: 10.1093/procel/pwaf066 (PMC12888918; doi:10.1093/procel/pwaf066)
Supplement: pwaf066_Supplementary_Figures_S1-S4 [file pwaf066_supplementary_figures_s1-s4.pdf]

Figure S1

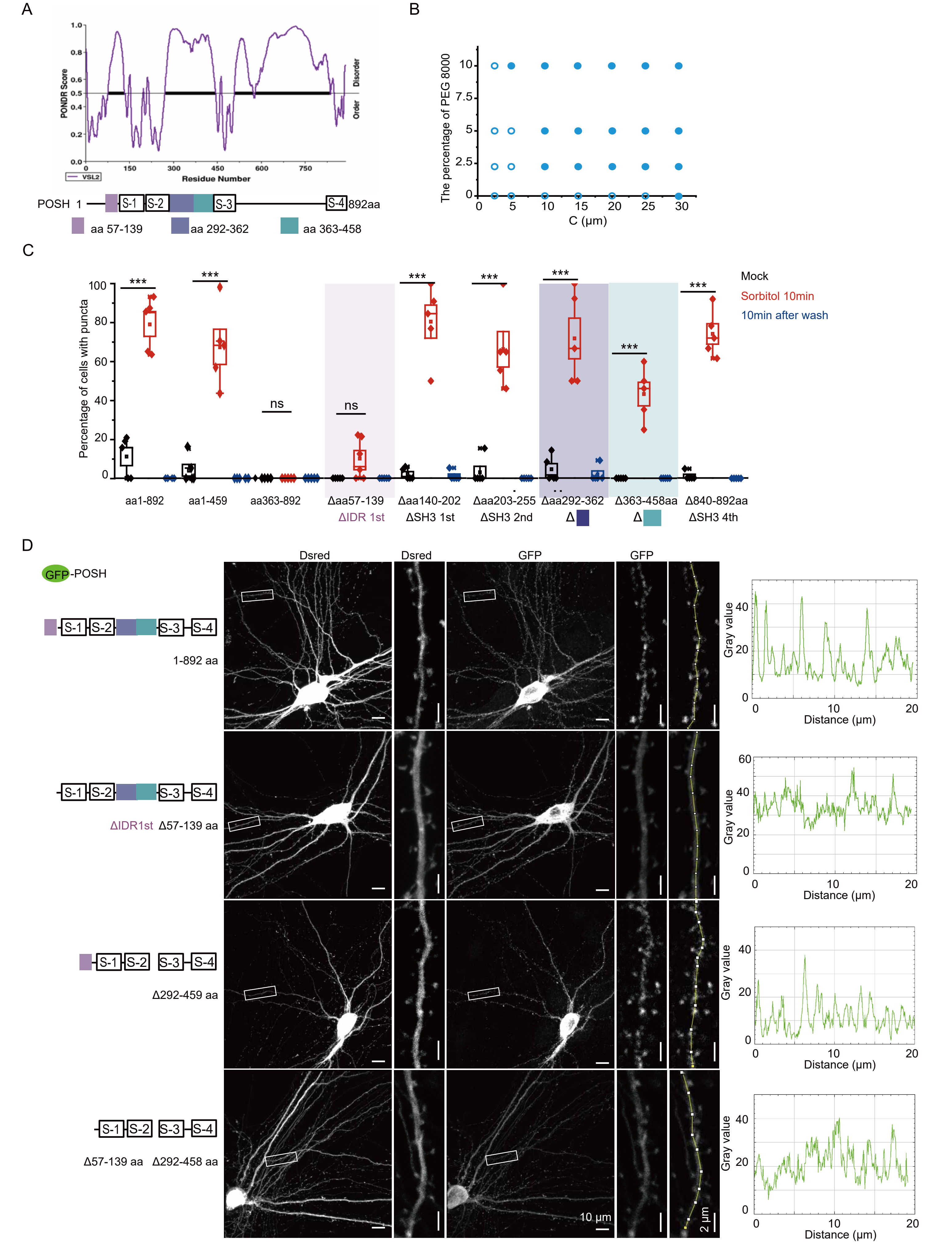

A
PONDR Score
Disorder
Order
Residue Number
VSL2
POSH 1
S-1
S-2
S-3
S-4
892aa
aa 57-139
aa 292-362
aa 363-458
B
The percentage of PEG 8000
C (μm)
C
Percentage of cells with puncta
Mock
Sorbitol 10min
10min after wash
***
ns
aa1-892
aa1-459
aa363-892
Δaa57-139
ΔIDR 1st
Δaa140-202
ΔSH3 1st
Δaa203-255
ΔSH3 2nd
Δaa292-362
Δ363-458aa
Δ840-892aa
ΔSH3 4th
D
GFP-POSH
Dsred
GFP
1-892 aa
ΔIDR1st Δ57-139 aa
Δ292-459 aa
Δ57-139 aa Δ292-458 aa
Gray value
Distance (μm)
10 μm
2 μm

Figure S1. Domain architecture of POSH governs phase separation in cellular and neuronal contexts

(A) Domain structure and intrinsic disorder score of POSH. The intrinsic disorder score of POSH was predicted using the PONDR prediction algorithm VSL2. Intrinsically disordered region (IDR) aa 57-139, aa 292-362, aa 363-458 were marked as indicated. S: SH3 domain. (B) Phase diagram showing POSH-PEG LLPS system. Purified POSH at varying concentrations was subjected to the droplet formation assay at room temperature in the present of indicated concentration of PEG 8000. The filled circles indicate where liquid-like droplets were observed, whereas the open circles indicate no droplet formation. The experiment was independently repeated three times with similar results. (C) Functional mapping of domains in POSH required for phase separation. Summary of puncta formation for GFP-POSH & mutants in ctrl & sorbitol treated HEK293T cells. Data means ± SEM; >200 cells per group. ***p<0.001; ns, not significantly different; one-way ANOVA with Tukey's test. Experiment repeated three times. (D) Puncta formation of POSH WT or its deletion mutants in hippocampal neurons. Left: Schematic representation of GFP-tagged POSH WT or deletion mutants. Middle: Representative images of hippocampal neurons transfected with the indicated constructs together with Dsred. Scale bars are 10 μm in the whole cell images and 2 μm in the magnified views of the boxed regions. Right: Line profiles of fluorescence intensities of GFP-tagged POSH constructs of the dashed line in the left panel. The experiment was independently repeated three times with similar results.

Figure S2

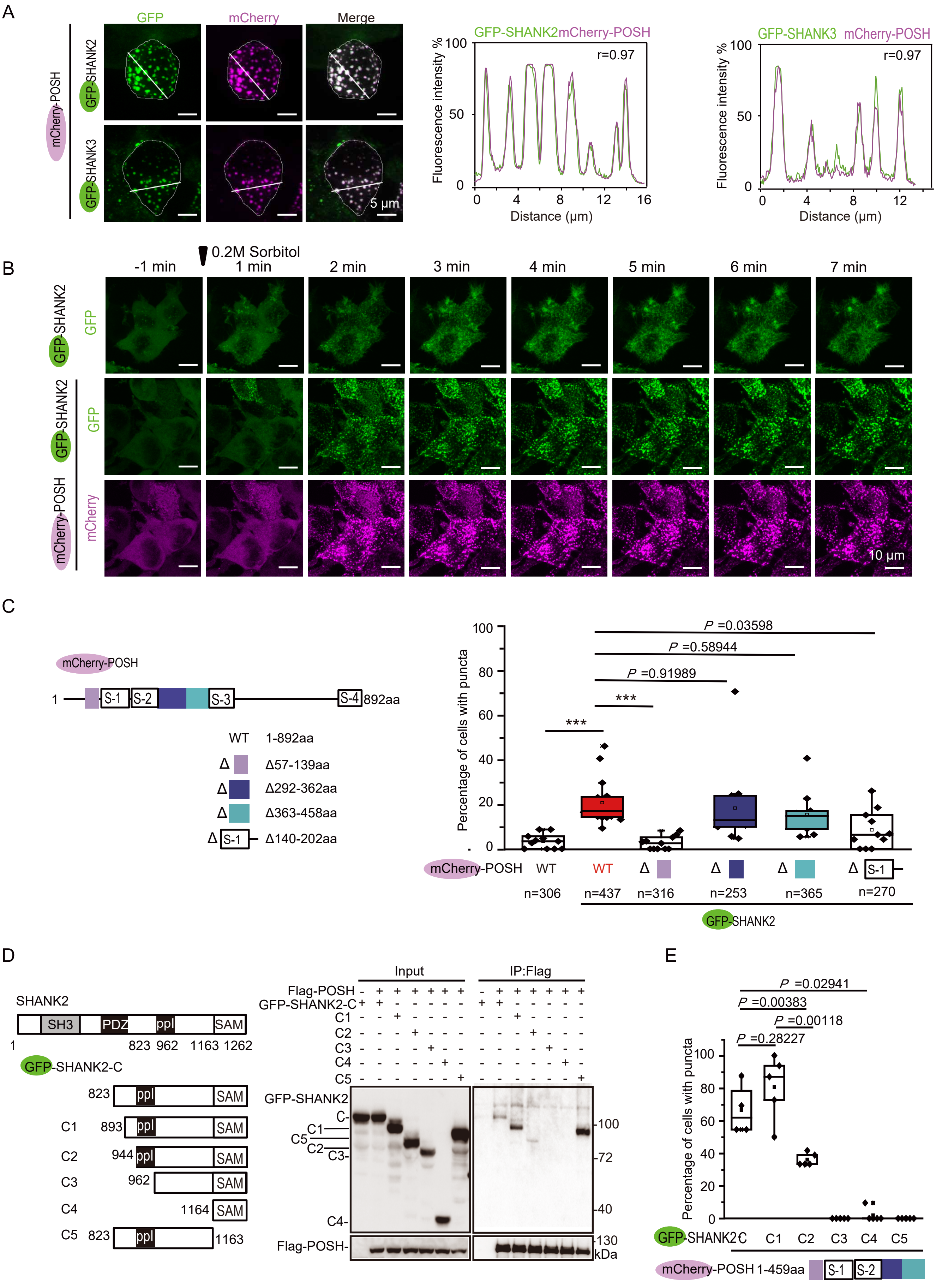
A
GFP
mCherry
Merge
mCherry-POSH
GFP-SHANK2
GFP-SHANK3
5 μm
GFP-SHANK2 mCherry-POSH
r=0.97
Fluorescence intensity %
Distance (μm)
GFP-SHANK3 mCherry-POSH
r=0.97
B
0.2M Sorbitol
-1 min
1 min
2 min
3 min
4 min
5 min
6 min
7 min
GFP-SHANK2
GFP
mCherry-POSH
mCherry
10 μm
C
mCherry-POSH
1
S-1
S-2
S-3
S-4
892aa
WT 1-892aa
Δ Δ57-139aa
Δ Δ292-362aa
Δ Δ363-458aa
Δ S-1 Δ140-202aa
Percentage of cells with puncta
P =0.03598
P =0.58944
P =0.91989
***
***
mCherry-POSH WT WT
n=306 n=437 n=316 n=253 n=365 n=270
GFP-SHANK2
D
SHANK2
SH3
PDZ
ppI
SAM
1 823 962 1163 1262
GFP-SHANK2-C
823
C1 893
C2 944
C3 962
C4 1164
C5 823 1163
Input
IP:Flag
Flag-POSH
GFP-SHANK2-C
C1
C2
C3
C4
C5
GFP-SHANK2
C-
C1
C5
C2
C3-
C4-
Flag-POSH-
100
72
40
130
kDa
E
P =0.02941
P =0.00383
P =0.00118
P =0.28227
Percentage of cells with puncta
GFP-SHANK2C C1 C2 C3 C4 C5
mCherry-POSH 1-459aa

Figure S2. Characterization of POSH/SHANKs co-condensation.

(A) HEK293T cells co-transfected with mCherry-POSH, GFP-SHANK2 or GFP-SHANK3 shown. Right: fluorescence intensity line profiles including Pearson's r. Scale bar, 5 μm. (B) Representative time-lapse images of HEK293T cells transfected with GFP-SHANK2 or co-transfected with mCherry-POSH under 0.2 M sorbitol treatment. Scale bars are 10 μm. (C) Mapping of domains in POSH required for co-condensation with SHANK2. Left: Schematic of mCherry-tagged POSH or deletion mutants. Right: Summary of puncta formation for mCherry-POSH WT or mutants co-expressed with GFP-SHANK2 in HEK293T cells. Data are presented as boxplots (centerline = median; box limits = (Q1, Q3); whiskers = max (minimum value, Q1-1.5×IQR), min (maximum value, Q3+1.5×IQR)); n = number of cells in the group. One-way ANOVA with Tukey's test. ***$p<0.001$ or as indicated. (D-E) Mapping of domains in SHANK2 required for co-condensation with POSH. Left: Schematic of GFP-tagged truncated SHANK2 proteins. Right: Co-IP assay for interaction of GFP-SHANK2-C or mutants with Flag-POSH. (E) Summary of puncta formation with GFP-SHANK2-C WT or mutants co-expressed with mCherry-POSH aa 1-459 in HEK293T cells. Data are presented as boxplots (centerline = median; box limits = (Q1, Q3); whiskers = max (minimum value, Q1-1.5×IQR), min (maximum value, Q3+1.5×IQR)). Group C: n=177; C1: 176 cells; C2: 144 cells; C4: 162 cells; C3, C5: no droplets. One-way ANOVA with Tukey's test. Experiments (A-E) were independently repeated three times.

Figure S3

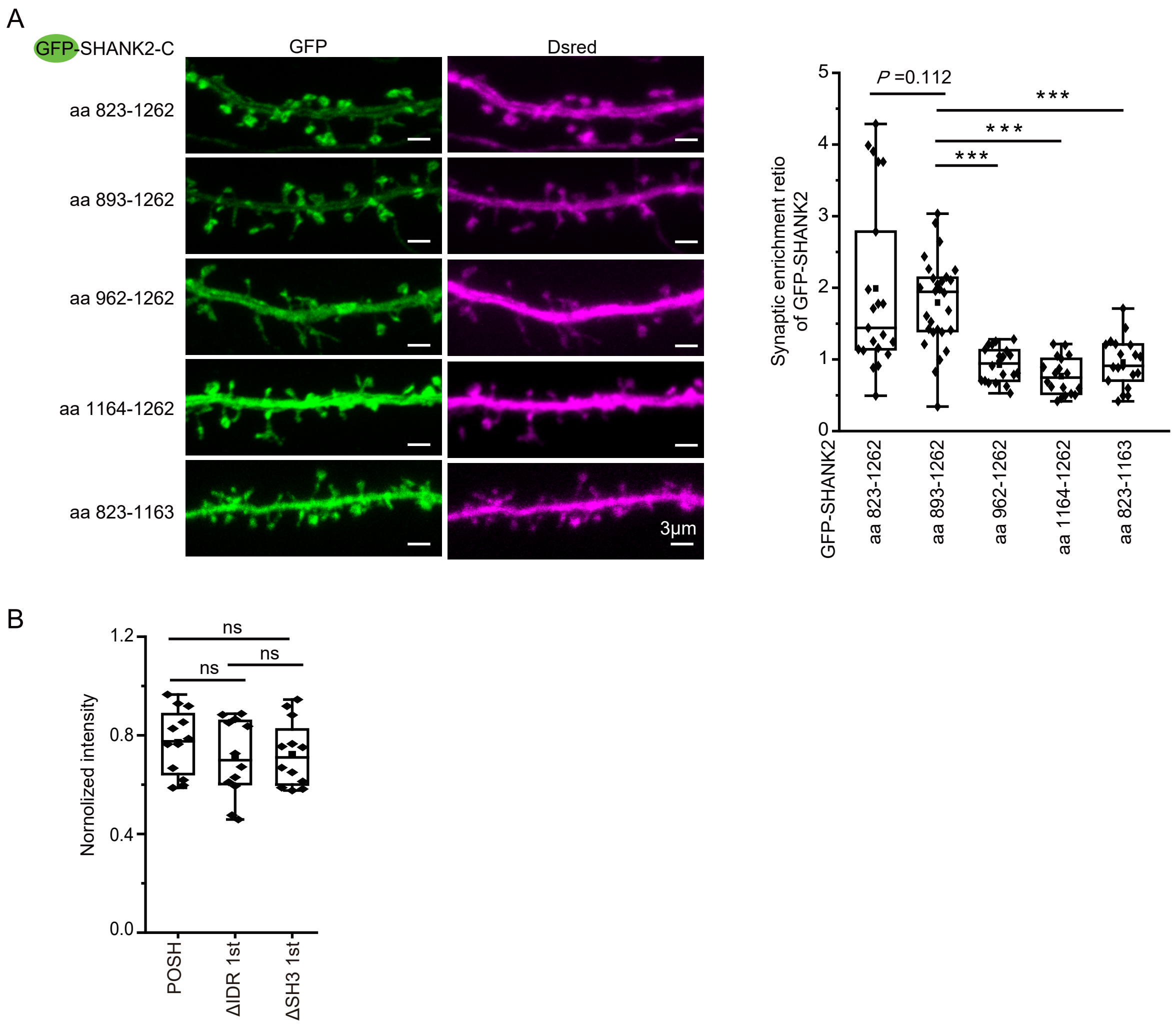

Figure S3. Assessment of Synaptic Targeting of GFP-SHANK2 Variants and Mutant POSH Expression Levels. (A) Hippocampal neurons were transfected with Dsred and GFP-SHANK2 C-terminal variants at DIV11 and imaged at DIV21. Quantification of imaging data shows synaptic targeting of GFP-SHANK2 variants. The synaptic enrichment ratio of GFP-SHANK2 is defined as ([GFP spine/GFP shaft]/[Dsred spine/Dsred shaft]). n≥18 dendrites from 12-16 neurons across 3 independent experiments per group. (B) Hippocampal neurons from POSH cKO mice were transfected with Dsred together with GFP-POSH, ΔIDR 1st, or ΔSH3 1st. Quantification of the intensity of GFP-POSH, ΔIDR 1st, or ΔSH3 1st normalized to Dsred intensity. n=12 neurons from 3 independent experiments per group. Scale bars = 3μm. (A-B) Data are presented as boxplots (centerline = median; box limits = Q1, Q3; whiskers = max (minimum value, Q1-1.5×IQR), min (maximum value, Q3+1.5×IQR)). ***p<0.001 or as indicated; ns, not significant. one-way ANOVA with Tukey's test.

Figure S4

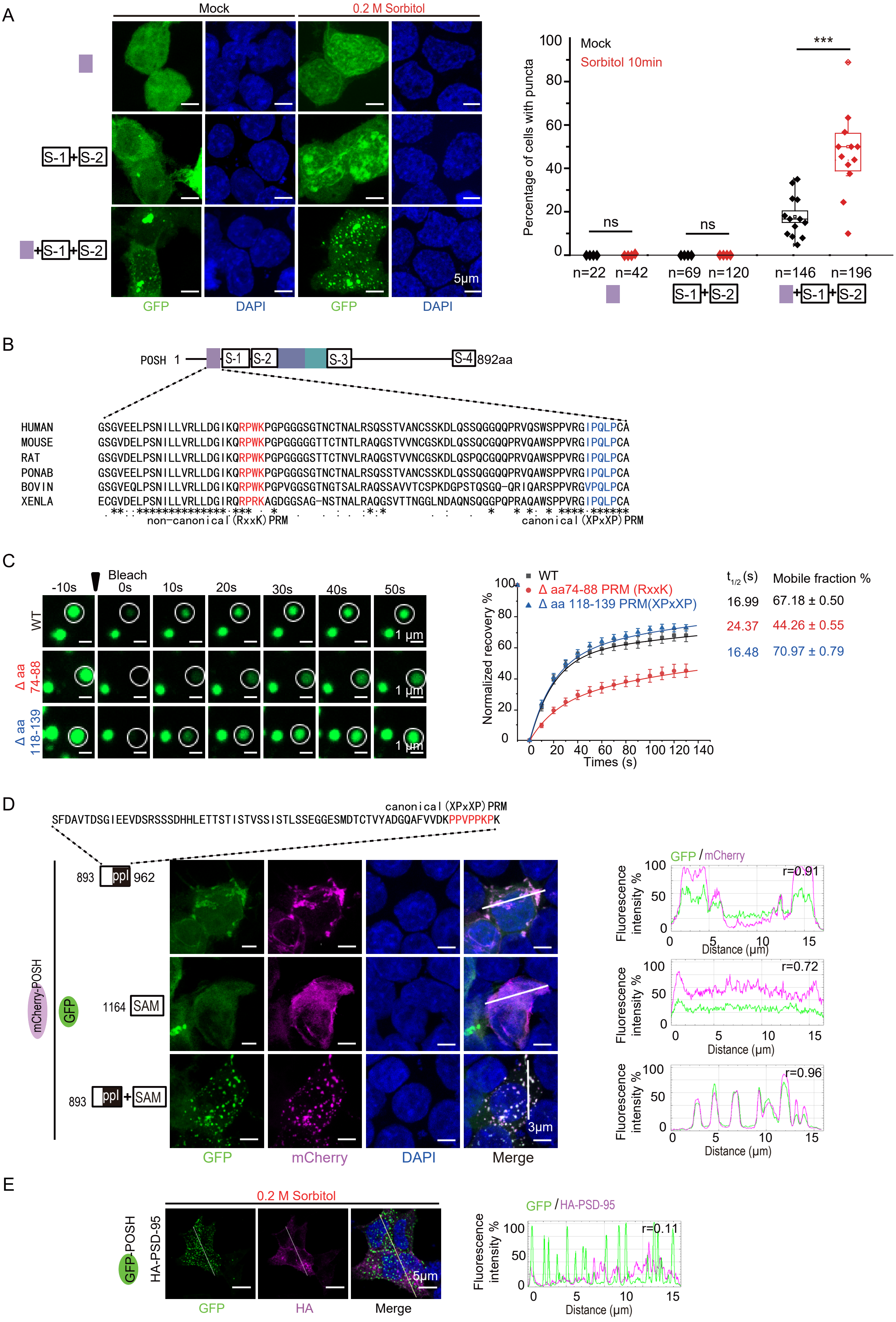
A
Mock
0.2 M Sorbitol
S-1 + S-2
+ S-1 + S-2
5μm
GFP
DAPI
GFP
DAPI
Mock
Sorbitol 10min
Percentage of cells with puncta
100
80
60
40
20
0
ns
ns
***
n=22 n=42
n=69 n=120
n=146 n=196
B
POSH 1
S-1 S-2 S-3 S-4 892aa
HUMAN GSGVEELPSNILLVRLLDGIKQRPWKPGPGGGSGTNCTNALRSQSSTVANCSSKDLQSSQGGGQPRVQSWSPPVRGIPQLPCA
MOUSE GSGVDELPSNILLVRLLDGIKQRPWKPGPGGGGTTCTNTLRAQGSTVVNCGSKDLQSSQCGQQPRVQAWSPPVRGIPQLPCA
RAT GSGVDELPSNILLVRLLDGIKQRPWKPGPGGGGSTTCTNVLRAQGSTVVNCGSKDLQSPQCGQQPRVQAWSPPVRGIPQLPCA
PONAB GSGVEELPSNILLVRLLDGIKQRPWKPGPGGGSGTNCTNALRSQSSTVANCSSKDLQSSQGGGQPRVQAWSPPVRGIPQLPCA
BOVIN GSGVEQLPSNILLVRLLDGIKQRPWKPGPVGGSGTNGTSALRAQSSAVVTCSPKDGPSTQSGQ-QRIQARSPPVRGVPQLPCA
XENLA ECGVDELPSNILLVRLLDGIRQRPRKAGDGGSAG-NSTNALRAQGSVTTNGGLNDAQNSQGGPQPRAQAWSPPVRGIPQLPCA
non-canonical (RxxK) PRM
canonical (XPxXP) PRM
C
-10s
Bleach 0s
10s
20s
30s
40s
50s
WT
Δ aa 74-88
Δ aa 118-139
1 μm
WT
Δ aa74-88 PRM (RxxK)
Δ aa 118-139 PRM(XPxXP)
Normalized recovery %
Times (s)
t1/2 (s) Mobile fraction %
16.99 67.18 ± 0.50
24.37 44.26 ± 0.55
16.48 70.97 ± 0.79
D
canonical (XPxXP) PRM
SFDAVTDSGIEEVDSRSSSDHHLETTSTISTVSSISTLSSEGGESMDTCTVYADGQAFVVDKPPVPPKPK
mCherry-POSH
GFP
893 ppl 962
1164 SAM
893 ppl + SAM
3μm
GFP
mCherry
DAPI
Merge
GFP / mCherry
Fluorescence intensity %
Distance (μm)
r=0.91
r=0.72
r=0.96
E
0.2 M Sorbitol
GFP-POSH
HA-PSD-95
5μm
GFP
HA
Merge
GFP / HA-PSD-95
Fluorescence intensity %
Distance (μm)
r=0.11

Figure S4. Mechanistic insights into POSH LLPS and its co-condensations with SHANKs.

(A) The LLPS of POSH was induced by the cooperation of its IDR 1st and tandem arrays of SH3 domains. The left panel illustrates the domain structure of the POSH chimera. Middle panel displays representative images of puncta formed by GFP-tagged POSH chimera with or without Sorbitol treatment. The right panel summarizes the puncta formation data for GFP-tagged POSH chimera. Here, n represents the number of cells from three independent experiments in each group. Data are presented as boxplots (centerline: median; box limits: Q1, Q3; whiskers: min/max within 1.5×IQR of Q1/Q3). ***$p<0.001$; ns, no significant difference; one-way ANOVA with Tukey's test. Scale bar, 5 μm. (B) Canonical and non-canonical proline-rich motifs (PRMs) in the sequence of POSH IDR 1st. A multiple sequence alignment of six representative POSH homologues reveals an exceptional degree of sequence identity between residues 57–139 of the mouse homologue. Sequence conservation is labelled beneath each amino acid position. (C) Representative images of GFP-POSH or mutants' condensates and quantification of fluorescence recovery from FRAP analysis. Data are presented as mean ± SEM (n = 25 puncta). The solid curve indicates a double exponential fit of the data. Key parameters, including recovery half-time ($t_{1/2}$) and mobile fraction, are shown on the right. Scale bars: 1 μm. (D) Representative images of puncta formed by co-expression of mCherry-POSH with GFP-SHANK2-C (aa 892-966), SAM, or aa892-966+SAM in HEK293T cells. The left panel shows the SHANK chimera diagram, the middle panel displays puncta images, and the right panel presents line profiles of fluorescence intensities and Pearson's correlation coefficient (r). The amino acid sequence diagram highlighting conserved PRMs in SHANK2/3 is also shown. Scale bars: 3 μm. (E) POSH co-condenses with SHANK2/3 but not PSD-95 in HEK293T cells. Left panel: Representative images of HEK293T cells co-transfected with GFP-POSH and HA-PSD-95, followed by sorbitol treatment. Right panel: Line profiles of fluorescence intensities and Pearson's correlation coefficient (r). Scale bars: 5 μm. All experiments were independently repeated three times with consistent results.

Materials and methods

Animals

The POSH flox/flox mouse model was generated by Beijing Biocytogen Co., Ltd. POSH cKO mice were obtained by crossing POSH flox/flox homozygous mice with Nestin-Cre mice (Jackson Laboratory, B6.Cg (SJL)-TgN(Nes-Cre)1Kln).

Primary neuron cultures

Hippocampal neuron cultures were prepared following our previously described procedures. Briefly, hippocampi from E16.5-E18.5 mouse embryos were dissected and dissociated with 0.125% trypsin in Hank's balanced salt solution without Ca^{2+} and Mg^{2+} for 15 min at 37°C. They were then seeded onto poly-L-lysine-coated coverslips in DMEM supplemented with 10% F12 and 10% FBS. The medium was changed 1 hour after plating to serum-free Neurobasal (NB, Gibco) medium supplemented with 2% B27 (Gibco) supplement and GlutaMAX (Gibco). Half of the medium was replaced with fresh medium every 4 days. Neurons were transfected with Lipofectamine 2000 (Gibco, Invitrogen) at 11-12 DIV and fixed with PFA at 20-21 DIV.

Constructs and protein expression

pCMS-EGFP-Flag-POSH, GST-POSH, and GFP-SHANK2/3 have been described previously. Various fragments and chimeric constructs of POSH were generated by standard PCR methods and individually inserted into pEGFP-C1 or mCherry-C1 vectors. Mutations or shorter fragments of SHANK2/3 (SHANK2: NM_001081370.3; SHANK3: NM_021423.4) were generated using standard PCR-based methods and cloned into pEGFP-C1. Vector sequences were confirmed by DNA sequencing.

To generate prokaryotic expression vectors, the cDNA encoding POSH was cloned into the pGEX-4T3 expression vector in frame with a GST tag. Recombinant proteins were expressed in Escherichia coli BL21 (DE3) cells at 30 °C for 4 hours to minimize protein degradation. N-terminal GST-tagged recombinant proteins were purified by a glutathione Sepharose affinity column. Eluted protein-containing samples were loaded onto an Amicon Ultra-4 Centrifugal Filter (Millipore) for concentration, and the buffer was replaced with 1X phosphate buffered saline (PBS), pH 7.2-7.4. When needed, recombinant protein tags were cleaved with Thrombin protease and separated by additional step of size-exclusion chromatography.

Protein labeling with chemical fluorophore

The chemical fluorophore iFluor 488 esters (AAT Bioquest) were dissolved in DMSO at a concentration of 10 mM and incubated with purified protein at room temperature for 1 hour (fluorophore to protein molar ratio was 1:1). The pH of the reaction buffer was adjusted to the range of 8.0-9.0 using 1 M pH 9.0 phosphate buffer. After being quenched with 0.2 M Tris, pH 8.0, the labeled protein was exchanged into the original storage buffer.

In vitro phase transition assay

Purified proteins were pre-cleared via high-speed centrifugation and diluted with buffer to design combinations and concentrations. For imaging assays, protein solutions were immediately loaded into a homemade glass-bottom chamber at room temperature. The chamber was imaged using a Zeiss LSM 800 with a 60× 1.40 N.A. oil objective lens. To capture droplet fusion events, images were taken every 1 second.

Fluorescence recovery after photobleaching assay

The FRAP assay was performed on a Zeiss LSM 800 confocal microscope with a 63× 1.4 N.A. oil objective. To quantify the FRAP of dynamically moving condensates, HEK293 cells transfected with GFP/mCherry - tagged constructs were placed under the microscope in 5% CO_2 at 37°C. Single-channel time-lapse imaging of GFP or mCherry was then performed in a single field using the continuous mode of the adaptive focus control system. After acquiring 5 frames as the 'Before' condition, circular regions within the condensates were photobleached with the laser at maximum intensity to achieve over 80% bleaching. This was followed by time-lapse imaging of 20 intervals as the 'After' condition. In each FRAP experiment, fluorescence intensities of a neighboring condensates with similar size to the one used for photobleaching was recorded for fluorescence intensity correction and a third region in the background with the same size was also recorded for background signal subtraction. Z-stack images were collected, and condensates that could not be tracked between 'Before' and 'After' were excluded. The fluorescence intensity difference between pre-bleaching and time 0 (immediately after photobleaching) was normalized to 100%, and the normalized intensity was used to plot the fluorescence recovery curve. The average fluorescence intensity of the last five time points was taken as the maximum recoverable proportion (mobile fraction) to calculate the half-recovery time ($t_{1/2}$). All experiments were completed within one hour of phase separation initiation and were repeated at least three times.

Cell culture and transfection

HEK293T cells were cultured in a 37 °C incubator supplied with 5% CO2. Transfection was performed using VigoFect according to the manufacturer's instructions (Vigorous).

Co-IP assay

For Co-IP assays, Flag-tagged POSH and GFP-tagged SHANK2/3 and their various mutants were co-expressed in HEK293T cells. After 24 hours of transfection, cells were lysed with buffer (50 mM Tris-HCl, pH 7.4, 100 mM NaCl, 2 mM EDTA, 1% (vol/vol) NP-40) supplemented with protease inhibitors. The lysates were then centrifuged at 12,000× g for 15 minutes at 4°C. The supernatants were incubated with anti-Flag M2 beads (Sigma-Aldrich) or for 2-4 hours at 4°C. After extensive washing, the captured proteins were eluted by boiling in SDS/PAGE sample buffer and detected by Western blot using specific antibodies.

Image acquisition and quantification

Confocal images were acquired using a Zeiss LSM 800 confocal microscope with a 60× 1.40 N.A. oil objective lens. For morphometric analysis of dendritic processes, GFP or Dsred was used as an unbiased cell filler. Secondary and tertiary dendritic branches with appropriate shaft width were selected for analysis. On average, between three and five branches were randomly selected to represent each neuron. For each branch, a length of approximately 60 μm was imaged, and all spines within this section were analyzed. Maximum image projections and three - dimensional projections were rendered with the Imaris Filament for spine density and volume calculations. Condensates were identified using Imaris Spots with sphericity > 0.8 per 10 μm dendrites for puncta quantification. For synaptic enrichment analysis, the fluorescence

intensity was quantified in the reconstructed shaft and spine channels. The synaptic enrichment ratio of SHANK2 was defined as ([SHANK2 spine/SHANK2 shaft]/[GFP or Dsred spine/GFP or Dsred shaft]). For quantification of SHANK3 clusters adjacent to Bassoon, two surface scenes (SHANK3 and Bassoon) were masked with Imaris XTension. A new channel was created from the overlapping regions and quantified per 10 μm dendrites. All morphological experiments were repeated at least three times with n ⩾12 for individual experiments. Images shown were z-projections of images taken at 0.2 μm step intervals and projected into a 2D image using a maximum intensity operation.

Quantification and statistical analysis

All data are presented as the mean ± SEM except for boxplots, which are depicted as centerline (median), box limits(Q_1, Q_3), whiskers [max(minimum value, Q_1-1.5×IQR), min(maximum value, Q_3+1.5×IQR)], where Q_1, Q_3, and IQR are the first quartile, third quartile, and interquartile range, respectively). The number of experiments is specified in the figure legends. For two-group comparisons, unpaired two-tailed Student's t-tests or Kolmogorov-Smirnov tests were employed. For multiple comparisons, one-way ANOVA with Tukey's post hoc test was used. All statistical analyses were conducted using Origin 8.0 software (OriginLab). Significance was defined as $p < 0.05$. In the figures, ** and *** denote $p < 0.01$ and $p < 0.001$, respectively.
